# Asthma Discordance in Twins Is Linked to Epigenetic Modifications of T Cells

**DOI:** 10.1371/journal.pone.0048796

**Published:** 2012-11-30

**Authors:** R. Scott Runyon, Leslie M. Cachola, Nitya Rajeshuni, Tessa Hunter, Marco Garcia, Regina Ahn, Fred Lurmann, Ruth Krasnow, Lisa M. Jack, Rachel L. Miller, Gary E. Swan, Arunima Kohli, Amanda C. Jacobson, Kari C. Nadeau

**Affiliations:** 1 Department of Pediatrics, Stanford University School of Medicine, Stanford, California, United States of America; 2 Sonoma Technology Inc., Petaluma, California, United States of America; 3 Center for Health Sciences, SRI International, Menlo Park, California, United States of America; 4 Division of Pulmonary Allergy and Critical Care Medicine, Columbia University College of Physicians and Surgeons, New York, New York, United States of America; Bellvitge Biomedical Research Institute (IDIBELL), Spain

## Abstract

T cells mediate the inflammatory responses observed in asthma among genetically susceptible individuals and have been suspected to be prone to epigenetic regulation. However, these relationships are not well established from past clinical studies that have had limited capacity to control for the effects of variable genetic predisposition and early environmental exposures. Relying on a cohort of monozygotic twins discordant for asthma we sought to determine if epigenetic modifications in T cells were associated with current asthma and explored whether such modifications were associated with second hand smoke exposures. Our study was conducted in a monozygotic twin cohort of adult twin pairs (n = 21) all discordant for asthma. Regulatory T cell (Treg) and effector T cell (Teff) subsets were assessed for levels of cellular function, protein expression, gene expression and CpG methylation within Forkhead box P3 (FOXP3) and interferon gamma-γ (IFNγ) loci. Comparisons by asthma and current report of exposure to second hand smoke were made. Treg from asthmatic discordant twins demonstrated decreased FOXP3 protein expression and impaired Treg function that was associated with increased levels of CpG methylation within the FOXP3 locus when compared to their non-asthmatic twin partner. In parallel, Teff from discordant asthmatic twins demonstrated increased methylation of the IFNγ locus, decreased IFNγ expression and reduced Teff function when compared to Teff from the non-asthmatic twin. Finally, report of current exposure to second hand smoke was associated with modifications in both Treg and Teff at the transcriptional level among asthmatics. The results of the current study provide evidence for differential function of T cell subsets in monozygotic twins discordant for asthma that are regulated by changes in DNA methylation. Our preliminary data suggest exposure to second hand smoke may augment the modified T cell responses associated with asthma.

## Introduction

Despite the rising rates of asthma prevalence and recent advancements in the use of genome-wide association studies, the mechanisms underlying the immunopathogenesis of asthma have yet to be elucidated. Substantial evidence suggests that several environmental exposures are important to the development of asthma. Past cohort studies have been limited by the ability to control for key confounders such as genetic predisposition and early environmental exposures [1]. Hence, studies among twins, monozygotic twins (MZT) in particular, have great potential to provide critical information on the role of genetic predisposition given the expected high concordance rate for asthma in twin pairs [2]. In addition to their identical genetic background, MZTs have similar childhood environmental exposures (i.e. diet, residential environment, maternal smoking, sibling order). The heritability of asthma has been reported to vary between 52–75% [3] leaving room for environmental contributions. Previous studies have identified childhood second hand smoke (SHS) as one of the environmental triggers associated with asthma [4], [5], [6], [7]. Although there is a strong link between SHS and asthma in adults and children [7], [8], [9], [10], the mechanisms are not well established; previous studies were not able to control for genetic makeup as we were able to do in the MZT cohort used in the current study.

Among the molecular changes suspected to be important in the development of asthma is altered regulation of CD4^+^CD25^high^CD127^low/−^ regulatory T cell (Treg) and CD4^+^CD25^neg^ effector T-cell (Teff) function, whereby the former contributes to airway immune tolerance and the latter to airway inflammation [11]. Our laboratory and others have reported that in asthmatic individuals such cellular mechanisms appear dysfunctional in T cells from bronchoalveolar lavage fluid (BAL) and peripheral blood (PB) [12]–[13]. Treg development and function appear to be strongly correlated with activation of the Forkhead box P3 (FOXP3) transcription factor, as low FOXP3 transcription and expression is consistently associated with reduced Treg function [14]. Control of FOXP3 expression is, in part, mediated by epigenetic modifications of the FOXP3 gene, including DNA methylation. For example, increased levels of methylation of CpG sites within the FOXP3 locus decreased expression of FOXP3 and subsequent Treg function [15], [16]. Recently, studies from our lab demonstrated a role for epigenetics in regulating asthma as increased CpG methylation of FOXP3 reduced lung function and increased asthma diagnosis [11]. Teff phenotype and function also appear to be under regulation of epigenetic modifications as methylation of key CpG sites within the IFNγ locus leads to decreased IFNγ expression within Teff, and skews Teff towards a T helper (Th) type 2 phenotype associated with asthma [12], [17]. Thus, epigenetic modifications of T cell subsets may influence the pathological mechanisms of asthma.

We hypothesized that functional impairment of Treg and Teff cells are due to epigenetic and molecular modifications in FOXP3 and IFNγ that are mechanistically linked to outcomes of asthma. To address this hypothesis, we conducted cellular and molecular studies at the functional, protein, transcript and epigenetic level of FOXP3 and IFNγ in purified T cell subsets among MZT discordant for asthma. We also explored whether report of exposure to SHS was associated with altered DNA methylation. Our investigation demonstrates that an increase in methylation of CpG sites within FOXP3 and IFNγ results in decreased protein expression and is associated with impaired T cell function that vary by SHS exposure and asthma diagnosis among MZT. Furthermore, the current study reports on the use of the candidate gene approach in epigenetic studies to trace the outcomes of a molecular change in a specific cell subtype to a functional consequence that is linked to a clinically significant health effect in humans.

## Methods

### Ethics Statement

The study was approved by the Stanford Institutional Review Board. Written informed consent was obtained by all participants or their parents (if <18 years of age) before participating in the study.

### Human Subjects

Twenty one pairs of monozygotic twins (MZTs) ranging 9 to 76 years old (average age 33) were recruited from the Twin Research Registry (TRR) at SRI International, Menlo Park, CA [18], [19], from Stanford University (Stanford, CA), and from Boston, Massachusetts. Our cohort consisted of MZT pairs discordant for asthma diagnosis (of which, six pairs were discordant for SHS exposure). Subjects were interviewed to determine medical history, disease onset and diagnosis, medication status, exposure to tobacco smoke and resident zip codes in the last 20 years. Patient demographics are summarized in Table 1. All subjects underwent pulmonary function testing (Koko Legend Spirometer). Asthma was defined by NHLBI criteria (EPR 2003). SHS exposure definition was used as per U.S. Surgeon General’s report (2006, 2010) and response to SHS questionnaire [20]. Subjects were excluded for any diagnosis of inflammatory condition (for example, viral illness or asthma exacerbation), obesity, allergy (for example, allergic rhinitis), autoimmune disease, cardiovascular disease, cancer, COPD or diabetes. No subject with a history of smoking, who currently smoked tobacco, or had *in utero* or childhood exposure to SHS was enrolled. Current exposure to SHS is defined as exposure in the last 5 years. No socioeconomic data were collected. None of the subjects that were sampled were on medications that were thought to be associated with modulating DNA methylation and/or T cell function (i.e. oral steroids or antiproliferatives).

**Table 1:** Subject demographics for twins discordant for asthma.

Variable	Asthmatic N (%)	Non-asthmatic N (%)
Age Mean (years)	33	33
Age Range (years)	9–76	9–76
Male	14 (33.3)	14 (33.3)
Total IgE*	154.8	21.6
FEV_1_ *	78.8	117.4
Asthma diagnosis		
Mild	7	1
Moderate	5	0
Severe	9	0
None	0	20
Recent second hand smoke exposure	6(14.2)	0 (0)

* Significant differences between asthmatic and non-asthmatic twins, P<0.05.

### Cell Isolation and Functional Assays

Treg and Teff were purified from up to 100 mL of peripheral whole blood (PB) from all subjects. All cell numbers were controlled for to decrease variability in comparison measurements between subjects. In addition, bronchoalveolar lavage (BAL) (n = 4 MZT pair) was collected from some subjects according to published procedures [16], [21]. CD4^+^ cells were isolated from each sample (Stem Cell Technologies) followed by staining and isolation of CD4^+^ CD25^neg^ Teff and CD4^+^CD25^hi^CD127^lo/−^ Treg subset populations to over 95% purity via FACS sorting (FACS Aria, BD Biosciences).

T cell functional assays were performed in round-bottom 96-well microtiter plates according to previously published methods [16] and further detail on these methods can be found in Data S1. Tetanus titers on subject plasma were determined using standard assays in the Stanford Hospital Clinical Laboratories with subject plasma and are expressed as units/mL.

### Flow Cytometry

Cells were fixed with Lyse/Fix PhosFlow buffer (BD Biosciences). For intracellular staining, fixed cells were permeabilized with Perm Buffer III (BD Biosciences) at 4°C for 30 minutes, followed by staining at 4° for 20 minutes. Flow cytometry was performed with an LSRII flow cytometer (BD Biosciences). Viable cells were identified with a live/dead probe (Invitrogen). Phenotypes of T cells were detected with antibodies against surface CD3, CD4, CD25, CD127 (BD Biosciences), and intracellular IFNγ (BD Biosciences) and FOXP3 (BioLegend) and stained per manufacturer’s recommended protocol.

### Quantitative PCR and Methylation Analysis

Total RNA or genomic DNA was isolated from purified Treg or Teff populations using the RNAeasy kit or the DNAeasy kit, respectively, according to the manufacturer’s protocol (Qiagen). For quantification of methylation within the FOXP3 and IFNγ loci, bisulfite modification of 1–2 µg of genomic DNA was performed using CpGenome Fast DNA modification kit (Chemicon International). Identification of the FOXP3 CpG loci of interest are based on our previous studies [21] and included a total of 13 CpG sites within the promoter and intronic region of FOXP3. For methylation analysis of IFNγ, five amplicons were amplified by using a HotStar Taq kit (Qiagen) and included a total of six CpG sites within the proximal promoter region of IFNγ. To display methylation data, we first obtained pyrosequencing data as to whether a particular CpG site was methylated (a threshold of 70% or higher methylation frequency was considered a methylated CpG site) in purified T cells that were controlled for cell number. Then, to calculate a separate % of CpG sites for a given locus, we divided the total number of methylated CpG sites (numerator) for a specific genetic locus (for example, either FOXP3 or IFNγ) by the total CpG sites sequenced (denominator) and used this % in our figures presented here. Further details on quantification of gene transcripts and measures of DNA methylation can be found in Data S1.

### Statistical Analysis

Laboratory data were assessed for normality and transformed if needed, before being subjected to appropriate statistical tests. Between-group means were compared with nonparametric Kruskal-Wallis ANOVA and pairwise post-test comparisons via the Dunn multiple comparison test (Graph Pad Prism Software 5.0; Prism Software). Sample size calculations based an increase in % CpG methylation of the FOXP3 locus in purified Treg cells by 40% showed that the study achieved adequate power (93%) with a 95% confidence interval using n = 42 subjects (n = 21 pairs discordant for asthma). Statistical differences in transcript, protein and CpG methylation levels between asthmatic and non-asthmatic twin pairs were assessed via the paired t-test. Linear regression was applied for comparison between FOXP3 transcript level and percentage of methylated CpG sites within FOXP3 and, separately, IFNγ. CpG sites with a methylation level that exceeded 70% was classified as methylated. For each sample, the percentage of individual CpG sites exhibiting methylation was compared, and fit without intercept using SAS v9.1 (SAS Institute, Cary, NC). The threshold for all statistical significance was set at a *p*-value <0.05. Geographic Information Systems were used analyzed by Sonoma Technologies using California Air Resources Board and EPA Air Pollution Monitoring Data (Dr. Fred Lurmann) to determine 1-year history (2010) exposure to fine particle PM_2.5_ and PM_10_ levels.

## Results

### Demographics

Twenty-one pairs discordant for asthma were analyzed. None of the non-asthmatic subjects had evidence of current smoking exposure in their home. Interestingly, of the subjects who were exposed to SHS, all had asthma as defined by NHLBI 2007 guidelines. As a group, asthmatic twins had increased total IgE as compared to the non-asthmatic twins (Table 1).

### Reduced T Cell Function in Asthmatic Twins

**Figure 1:**
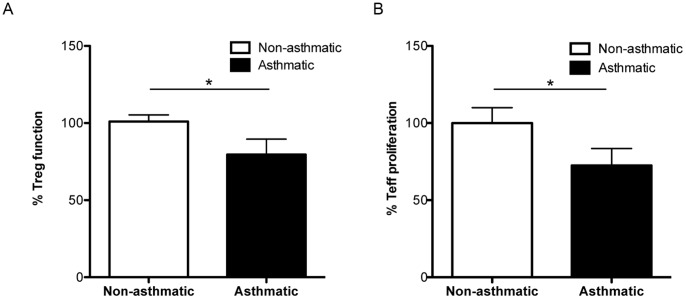
Differential T cell function between asthmatic and non-asthmatic twins. Function of purified Treg (A) and purified Teff (B) from non-asthmatic twins (n = 21, white bars) and their asthmatic (n = 21, black bars) twin partner was assessed via ^3^H-thiymidine incorporation. A) Suppressive function of purified CD4+CD25+ Treg to CD4+ CD25neg Teff is presented as % function. B) Proliferation of purified CD4+ Teff to tetanus antigen. Data are presented as mean ± SD, * p<.05.

To address the hypothesis that functional impairment of Treg and Teff cells are due to molecular modifications in FOXP3 and IFNγ that are linked mechanistically to outcomes of asthma, we first determined the extent to which Treg and Teff cell function was impaired in the MZT with asthma vs. the paired MZT without asthma. Our laboratory has previously published on the presence of dysfunctional Treg in the PB and bronchoalveolar lavage fluid (BAL) of asthmatic subjects using standard proliferation assays [16]. We tested the suppressive activities of Treg against autologous responder Teff and found that function of PB Treg from asthmatic twins was significantly lower when compared to non-asthmatic twins (Figure 1A, p<.05). Bronchoalveolar lavage fluid (BAL) Treg function was not analyzed due to limited number of Treg available for functional assays. In parallel, function of Teff from twin pairs discordant for asthma was assessed by proliferation assays to tetanus antigen. Teff from asthmatic twins showed reduced proliferation to tetanus antigen compared to non-asthmatics in our twin cohort (Figure 1B, p<.05). Tetanus Ig titers were not significantly different between asthmatic and non-asthmatic twins (Figure S1). These ex-vivo data suggest that Treg and Teff immune function is reduced in asthmatics.

### Lower Expression of FOXP3 and IFNγ in Treg and Teff, Respectively, in Asthmatics

**Figure 2:**
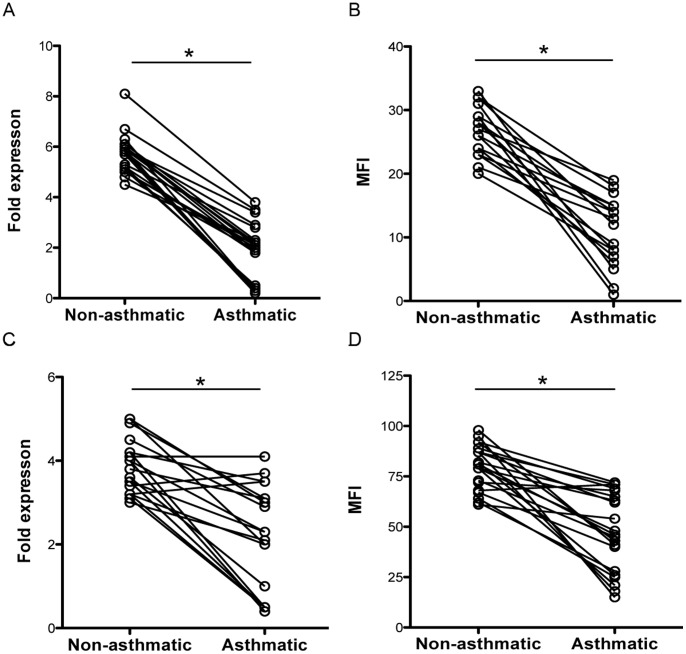
Reduced expression of proteins involved in Teff and Treg function during asthma. A) FOXP3 gene expression and B) FOXP3 protein was assessed from purified Treg from asthmatic and non-asthmatic twin pairs. In C) IFNγ gene expression and D) IFNγ protein levels were assessed from purified Teff cells from asthmatic and non-asthmatic twin pairs. Gene expression was determined by QT-PCR and is shown as relative fold expression of candidate genes to expression of the housekeeping gene β-glucuronidase. Protein levels were determined by intracellular flow cytometry and shown as mean fluorescence intensity (MFI). Data points represent individuals and twin pairs are connected via lines, * p<.001.

Since we observed decreased Treg function in asthmatic twins (Figure 1), we next assessed the relative levels of FOXP3 protein and transcription in Treg from MZT discordant for asthma. We found that the asthmatic twins had significantly lower expression of FOXP3 as compared to Treg from their non-asthmatic twin (Figure 2A, p<.001). Reduced transcript levels were associated with reduced FOXP3 protein levels in Treg from asthmatic twins as determined by intracellular flow cytometry (Figure 2B, p<.001).

IFNg is one of the key cytokines produced by Th1 CD4^+^ Teff cells. As we also observed decreased Teff function from asthmatic twins, we wanted to assess whether IFNγ expression varied by asthma. Therefore, we measured the protein levels and gene transcripts for IFNγ from peripheral blood Teff. Teff from asthmatic twins had reduced transcript expression and significantly decreased IFNγ protein expression as compared to non-asthmatic twins (Figure 2C and D, p<.001). These data indicate that proteins and gene transcripts known to be important for Teff (IFNγ) and Treg (FOXP3) function are modified during asthma in this MZT cohort.

### Epigenetic Modification of T Cells during Asthma

**Figure 3:**
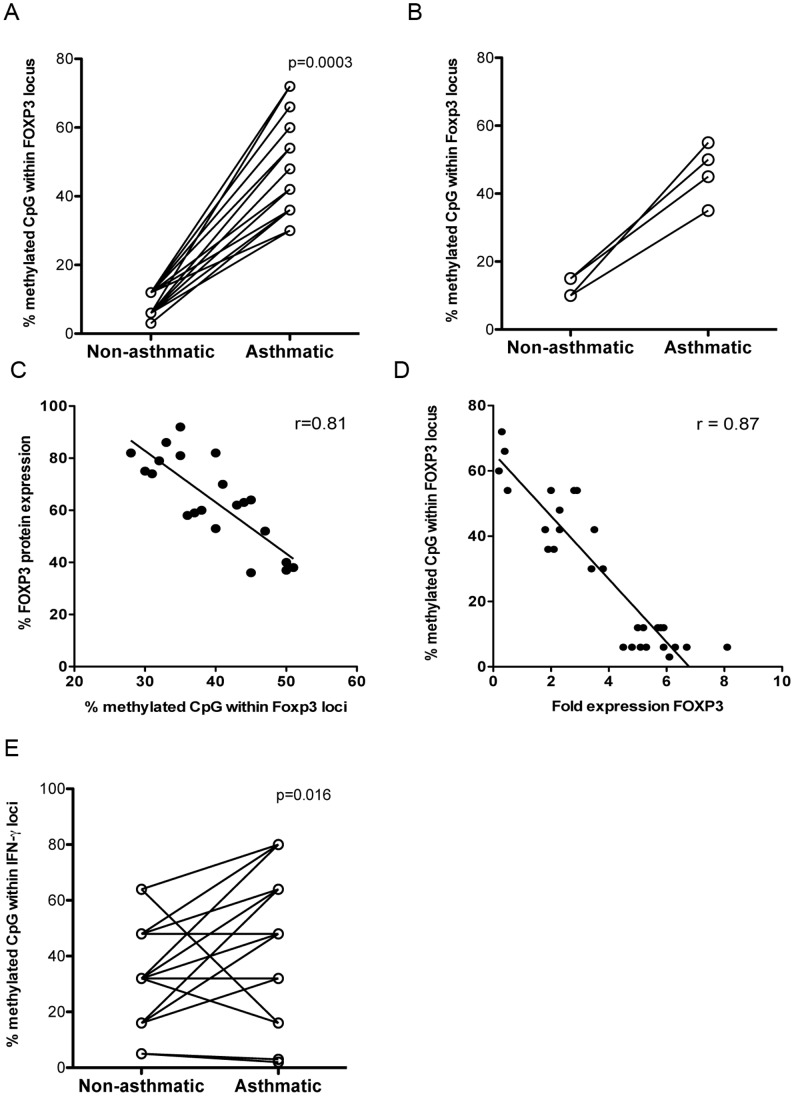
Epigenetic modification of FOXP3 and IFNγ during asthma. Treg were purified from the PB (A, n = 21) or bronchoalveolar lavage (BAL) (B, n = 4) of MZT pairs discordant for asthma and methylation of 13 CpG sites within the FOXP3 locus was quantified. A CpG site was considered methylated after exhibiting methylation at that CpG site in ≥70% of the pyrosequencing reactions. Linear regression and Spearman’s correlation analysis of C) FOXP3 protein and % of methylated CpG sites within FOXP3(% calculated as number of methylated CpG sites divided by total number of CpG sites in the FOXP3 locus) or D) FOXP3 transcript (fold expression) and % of methylated CpG sites within FOXP3. E) Teff were purified from asthmatic and non-asthmatic MZT pairs (n = 21) and methylation of 6 CpG sites within the IFNγ locus was quantified. Data points represent individuals and twin pairs are connected via lines, r =  correlation coefficient.

Previous studies have demonstrated that epigenetic modifications in CpG-rich regions within the FOXP3 locus are associated with stable FOXP3 expression [15], [22], [23]. Because we observed differential levels of FOXP3 transcript between MZT pairs discordant for asthma, we next determined whether changes in FOXP3 expression were associated with altered DNA methylation. Using pyrosequencing technology, we assessed the methylation status of 13 CpG sites within FOXP3 and found that CpG sites within FOXP3 from purified Treg were roughly six times as methylated in the asthmatic MZT vs. the non-asthmatic MZT (Figure 3A, p  = .0003). Similar results were found in Treg purified from bronchoalveolar lavage fluid (BAL) from a limited number of twin pairs (Figure 3B). Linear regression and Spearman’s correlation analysis between levels of FOXP3 protein and FOXP3 CpG methylation demonstrated an association between decreased CpG methylation and higher FOXP3 expression in Treg (Figure 3C, r  = 0.81). Similar analysis of the levels of FOXP3 transcript and FOXP3 CpG methylation demonstrated an association between decreased CpG methylation and higher FOXP3 expression in Treg (Figure 3D, r  = 0.88). These data suggest that there is differential epigenetic regulation of FOXP3 in MZT pairs discordant for asthma.

To determine if epigenetics were involved in the observed differences in IFNγ expression in Teff from the asthmatic twins, we also assessed CpG methylation of six CpG sites in the IFNγ locus. Similar to our FOXP3 data, we observed an increase in the percentage of CpG sites methylated within the IFNγ locus in asthmatic Teff as compared to Teff from their non-asthmatic twin (Figure 3E, p  = 0.016). Due to unavailability of IFNγ methylation primers at the time bronchoalveolar lavage fluid (BAL) cells were collected, bronchoalveolar lavage fluid (BAL) Teff were not assessed for methylation of CpG sites within the IFNγ locus. Collectively, the data in Figure 3 suggest that T cells are modified at a DNA level during asthma.

### Modifications of FOXP3 and IFNγ are Associated with Report of Current SHS among Asthmatics

**Figure 4:**
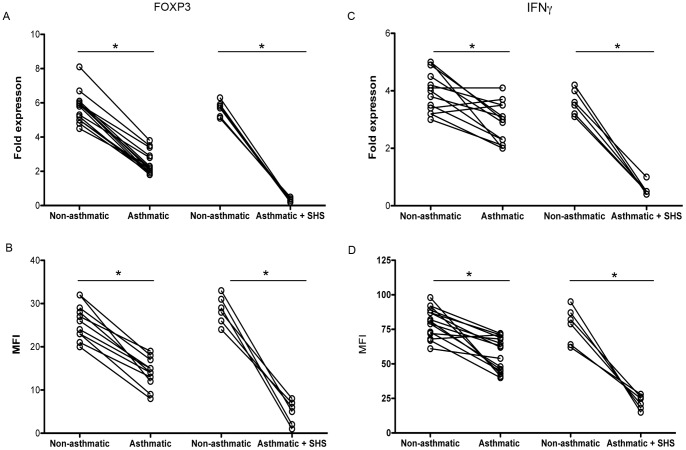
Reduction of FOXP3 and IFNγ is augmented with recent SHS exposure. Peripheral blood Treg or Teff (CD4+CD25neg) were purified from twin pairs discordant for asthma and concordant for no SHS exposure (n = 15) and discordant for both asthma and SHS exposure (n = 6). In A and B, Treg were assessed for FOXP3 transcript (A) and FOXP3 protein (B). In C and D, Teff were assessed for IFNγ transcript (C) and protein (D). Gene expression was determined by QT-PCR and is shown as relative fold expression of candidate genes to expression of the housekeeping gene β-glucuronidase. Protein levels were determined by intracellular flow cytometry and shown as mean fluorescence intensity (MFI). Data points (circles) represent individuals and individual twin pairs are connected via a line, p<.05 for non-asthmatic vs. asthmatic or asthmatic + SHS for all groups.

Several of the asthmatic twins demonstrated very low levels of FOXP3 and IFNγ expression (Figure 2), and we were interested in determining if environmental SHS affected T cell protein expression and function in these asthmatic twins. Additionally, epigenetic modifications of DNA are, in part, mediated by SHS [24], [25]. Thus, we further explored if known recent (in the last 5 years) SHS exposure was associated with the modified gene expression and CpG methylation differences we observed between the asthmatic vs. non-asthmatic MZT twin pairs. When FOXP3 expression was measured, we found that FOXP3 expression was the lowest in Treg from the 6 asthmatic twins with current SHS exposure as compared to asthmatics without SHS or non-asthmatic twins (Figure 4A, p<.05). A parallel decrease in FOXP3 protein levels in Treg from asthmatic twins with current SHS exposure was observed (Figure 4B, p<.05).

**Figure 5:**
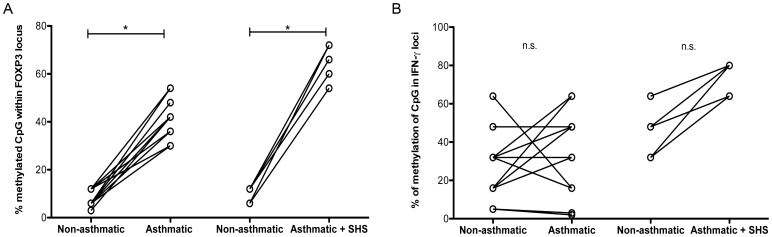
Increased epigenetic modification of T cells with current SHS exposure during asthma. Peripheral blood CD4+CD25+ Treg or CD4+CD25neg Teff were purified from twin pairs discordant for asthma and concordant for no SHS exposure (n = 15) and discordant for both asthma and SHS exposure (n = 6). Genomic DNA was sequenced for methylation of CpG sites within A) FOXP3 or B) IFNγ. *p<.05, n.s.: not significant.

We also assessed Teff from the six MZT pairs discordant for both asthma and SHS exposure for levels of IFNγ. As shown in Figure 4C and D, both IFNγ transcript level and protein expression is decreased further in asthmatic twins with current SHS exposure as compared to their non-asthmatic twin or asthmatic twins without current SHS exposure (Figure 4, p<.05). In addition, we assessed the level of CpG methylation of FOXP3 and IFNγ in Treg and Teff, respectively, from twins with recent SHS exposure vs. those twins with no recent SHS exposure. Our data demonstrate a significant increase in the level of methylation of CpG sites within FOXP3 loci with recent SHS exposure as compared to individuals with no recent SHS (Figure 5A, p<.05). Linear regression and Spearman’s correlation analysis of FOXP3 transcript and CpG methylation within the FOXP3 locus of Treg from asthmatic subjects either with or without SHS are presented in Figure S2. In addition, we also examined two pairs of twins that were concordant for SHS exposure but with no asthma (FigureS3). In a small (n = 4) sample set, we found that these twins had some, but not full, CpG methylation in the FOXP3 locus (mean 34%: range 31–38%) vs. twins with no asthma and no SHS exposure (mean 7%: range 3–12%).

The amount of methylation of the IFNγ locus in Teff from asthmatics with SHS tended to be higher than that in Teff from asthmatics without SHS or non-asthmatics, but these data did reach significant values (Figure 5B). These data indicate that recent SHS exposure may modify T cells at the DNA level and augment the reduced FOXP3 and IFNγ expression occurring in T cells during asthma.

### Impairment of Teff Function and Ig Production in Asthmatics with Recent SHS

**Figure 6:**
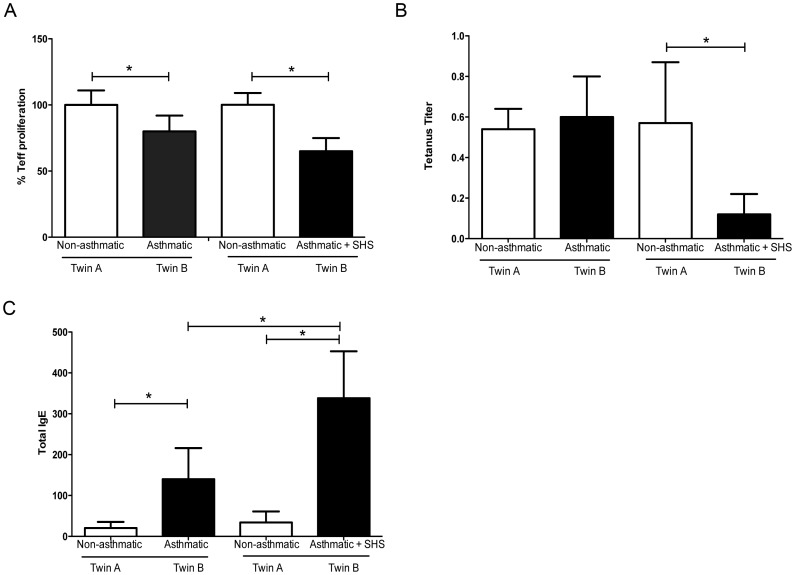
Decreased immune function in asthmatics with SHS. Immune parameters from MZT pairs discordant for asthma (n = 15 non-asthmatic (Twin A) and asthmatic (Twin B)) or MZT pairs discordant for both asthma and SHS (n = 6 non-asthmatic (Twin A) and asthmatic + SHS (Twin B) were assessed. A) Teff proliferation to tetanus antigen. B) Tetanus Ig titers. C) Total serum IgE titers. Data are presented as mean ± SD, *p<.05.

The impaired IFNγ production observed in asthmatic patients with recent SHS led us to test whether Teff function in subjects with SHS in our MZT cohort was further impaired. Proliferation of Teff to tetanus antigen was reduced in asthmatic twins with SHS as compared to their non-asthmatic twin (Figure 6A, p<.05). Ig production can be indicative of both T and B cell function. We additionally assessed tetanus-specific Ig in asthmatics with SHS and found that tetanus Ig titers were significantly reduced in the asthmatic twins with SHS as compared to their non-asthmatic twin partners (Figure 6B, p<.05). These data suggest that Teff and B cell function likely are affected by environmental SHS.

Treg exert a direct effect on B cells, suppressing the production of allergen-specific IgE and inducing IgG4 [26]; reduced FOXP3 expression, and subsequent reduction in Treg function, has been implicated with elevated IgE levels in both human and mouse studies [22], [27], [28], [29]. To determine if there was an association between Treg function and IgE, we quantified total serum IgE levels in our MZT cohort. Figure 6C shows that asthmatics with SHS had increased total IgE as compared to asthmatics with no SHS (p<.05). Collectively, the *ex-vivo* functional data presented in Figure 6 suggest that SHS impairs T cell, and possibly B cell, function in asthmatics and further suggest that these impairments are due to environmental and not genetic influences.

## Discussion

Data presented in this study demonstrated an association between increased CpG methylation and decreased FOXP3 and IFNγ expression in Treg and Teff that was linked to cellular dysfunction. This is the first study to our knowledge that provides data from MZT to trace plausible mechanistic pathways from molecular events (i.e. DNA methylation of CpG sites) in specific genes (i.e. FOXP3 and IFNγ) to transcript, to protein, to cellular changes and then to clinical outcomes and immune function (specifically, total IgE levels and tetanus response). Moreover, T cell modifications of FOXP3 and IFNγ were amplified in the MZT with recent SHS vs. the paired MZT without recent SHS.

As shown in previous studies, CpG methylation can be induced through epigenetic mechanisms and triggered via environmental exposures [16], [30], [31]. A recent study demonstrated that buccal mucosa cells from children exposed to maternal smoking *in utero* had differences in global and gene-specific DNA methylation, and that the long-term effects of *in utero* tobacco smoke exposure may be mediated through DNA methylation [24], [25]. We were interested in the effects of recent SHS exposure (in the last 5 years) and did not include subjects with a history of smoking or a history of childhood/*in utero* exposure or who currently smoked. Epigenetic changes have been shown to affect FOXP3 and IFNγ expression and mutations and/or deficiencies in FOXP3 or IFNγ can result in development in autoimmune disease, infections or asthma [30], [32]. The methylation data in our study were not unique to one tissue region but were found to be different in some discordant pairs in both the PB and BAL, suggesting similar immunological mediator function at different tissue sites. However, increased numbers of subjects in studies focused on CpG methylation in specific cells and tissues are necessary to be able to further determine mechanistic pathways that link a molecular event to disease outcome. Even though we observed decreased IFNγ gene and protein expression in asthmatic twins with SHS, there was not a significant increase in methylation of the six CpG sites we analyzed. Additional studies will include the assessment of all CpG methylation sites within the IFNγ locus. Finally, further detailed mechanistic studies on the correlation between DNA methylation of FOXP3 and IFNγ in Treg and Teff, respectively, and disease state are warranted.

As DNA methylation has been associated with age, a potential confounder in our study is age of each twin pair. Previous studies have elegantly demonstrated that certain genes are either hyper- or hypo-methylated with age in MZTs however, these studies did not identify DNA modification of FOXP3 or IFNγ with respect to age in different T cell subsets [33], [34], [35]. When we assessed age and CpG methylation of either FOXP3 or IFNγ in purified T cell subsets in our twin cohorts to date we did not find an association; however, further studies with twin subjects at different age ranges would be needed to optimally determine associations between age (neonatal to geriatric) and CpG methylation of FOXP3 or IFNγ. So far to date, our analysis shows that, even within the same twin pair (i.e. naturally controlled for age) there are statistically significant differences in methylation events.

The effects of SHS on the immune response are an important, but understudied, area of focus. To fully test the interaction of environmental exposures between genetic predisposition and disease outcomes of asthma, broader studies including concordant and discordant twins for SHS with and without asthma are needed. However, our preliminary data suggest that recent SHS induces epigenetic modifications in IFNγ and FOXP3 in Teff and Treg populations, respectively, and results in impaired cellular function.

In choosing an approach that included MZT, we were able to eliminate confounding factors such as genetic background, in utero exposure, major childhood exposures, age, and sex. There is evidence emphasizing the genetic heritability of asthma [13]; however, environmental factors play an important role in mediating epigenetic events that are also associated with disease outcome. In this study, we began to investigate potential effects of the environmental factors outside of SHS, for example, air pollution, on Teff and Treg function. Although a geographical information system (GIS) analysis of our cohort thus far has yielded no significant differences in PM2.5 and PM10, these data analyses were limited to n = 10 pair of discordant asthma twins; therefore, we will continue to examine correlations between GIS and DNA methylation in the MZT.

We recognize that more twins with SHS exposure and no asthma are needed. Moreover, studying twin pairs longitudinally will enable a better understanding of the possible molecular linkage between lung disease and methylation of CpG sites in FOXP3 and IFNγ in Treg and Teff, respectively.

Our data demonstrated an association between Treg impairment and total IgE (Figure 6C). In both mice and human, impaired Treg function is associated with increased IgE and atopic conditions. Scurfy mice, which present a deletion in Foxp3, have impaired capacity to generate natural Treg, or mice depleted of Foxp3+ Treg have elevated IgE levels [28], [36]. In humans, single nucleotide polymorphisms of FOXP3 are associated with atopic pathologies in childhood, including increased IgE levels [27]. Additionally, the rare disorder of the immune system, immune dysregulation, polyendocrinopathy, enteropathy X-linked (IPEX) syndrome, is caused by mutations in the FOXP3 gene that result in defective development of CD4+ CD25+ Treg; children with IPEX syndrome demonstrate high levels of serum IgE (reviewed in [37]).

As Treg are major components in both the induction and the maintenance of tolerance, interfering with Treg function (at the DNA level as observed in our study) could affect responses downstream of any Treg function (i.e. production of TGFβ or IL10, inhibition of Th2 cells and dampening of inflammatory responses). The data on T cell modifications induced by SHS we present in this study may influence multiple arms of the immune system including B cells, T cells, mast cells and NK cells.

In conclusion, our study demonstrated a reduction in FOXP3 and IFNγ expression and Treg and Teff function in asthmatic MZT associated with epigenetic changes in the FOXP3 and IFNγ genetic loci. In addition, our data suggest that recent SHS may have a significant effect on methylation levels in these genes and subsequent impairment of cellular function of T cells. Through data presented in the current study, we begin to address the epigenetic consequences of exposure to SHS, and how these in turn are associated with asthma. The two key unique elements to this study that enabled us to begin to fill this gap include 1) enrollment of MZTs, including those discordant for exposure to SHS and discordant for asthma, to eliminate genetic predisposition and differing *in utero* and early environmental exposures as confounders, and 2) focus on molecular events that likely vary by T cell type. Our data so far suggest that epigenetic modifications may be cell specific. This unique approach and these two key elements have begun to overcome limitations from past studies. Our study represents an important approach in epigenetic studies since we use hypothesis-driven, candidate genes to link outcomes of a molecular change in a specific cell subtype to a functional consequence that could lead to a clinically significant health effect in humans.

## Supporting Information

Figure S1 Tetanus Ig titers in MZT pairs discordant for asthma. Non-asthmatic twins (white bar) vs. asthmatic twins (black bar).
(TIF)

Figure S2 Linear regression analysis of FOXP3 expression and CpG methylation within the FOXP3 locus of asthmatics. A) Asthmatic MZT subjects with no SHS (n = 15) and B) All asthmatic MZT subjects with SHS exposure (n = 6).
(TIF)

Figure S3 FOXP3 CpG methylation analysis in non-asthmatic MZT pairs with SHS (n = 2).
(TIF)

Data S1 (DOC)
