# Supplementary figures and images for: Asthma Discordance in Twins Is Linked to Epigenetic Modifications of T Cells
Source: PLoS One. 2012 Nov 30;7(11):e48796. doi: 10.1371/journal.pone.0048796 (PMC3511472; doi:10.1371/journal.pone.0048796)

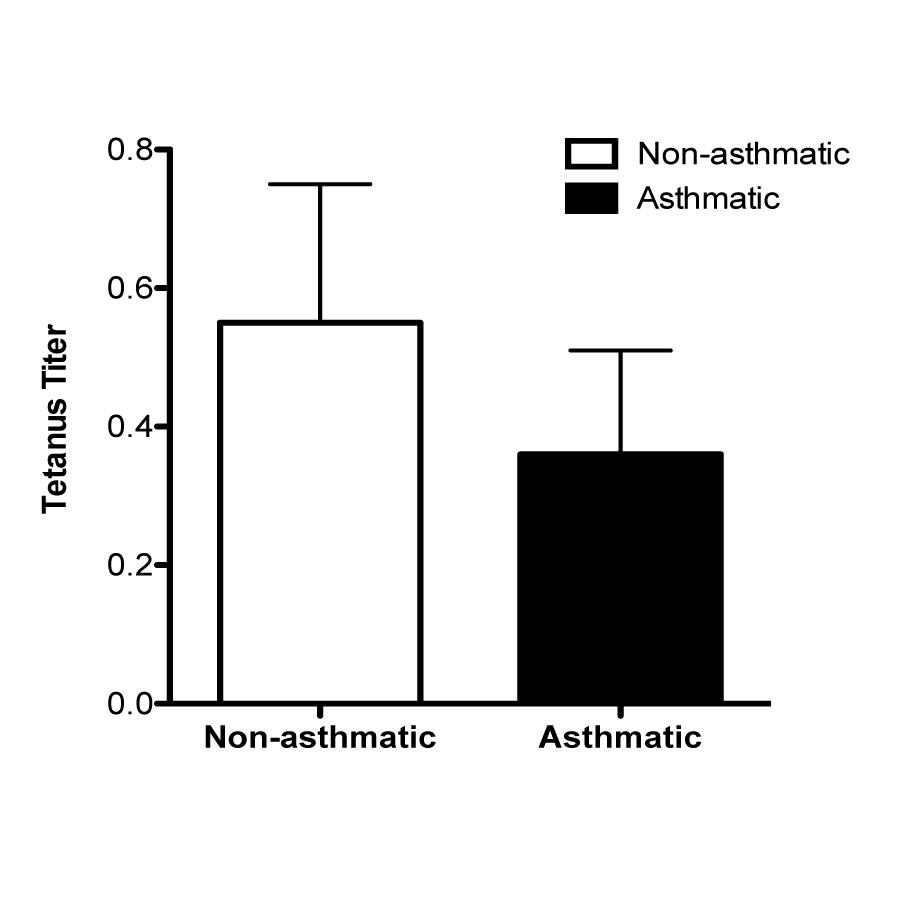

Supplement: Figure S1 — Tetanus Ig titers in MZT pairs discordant for asthma. Non-asthmatic twins (white bar) vs. asthmatic twins (black bar). (TIF) [file pone.0048796.s001.tif]

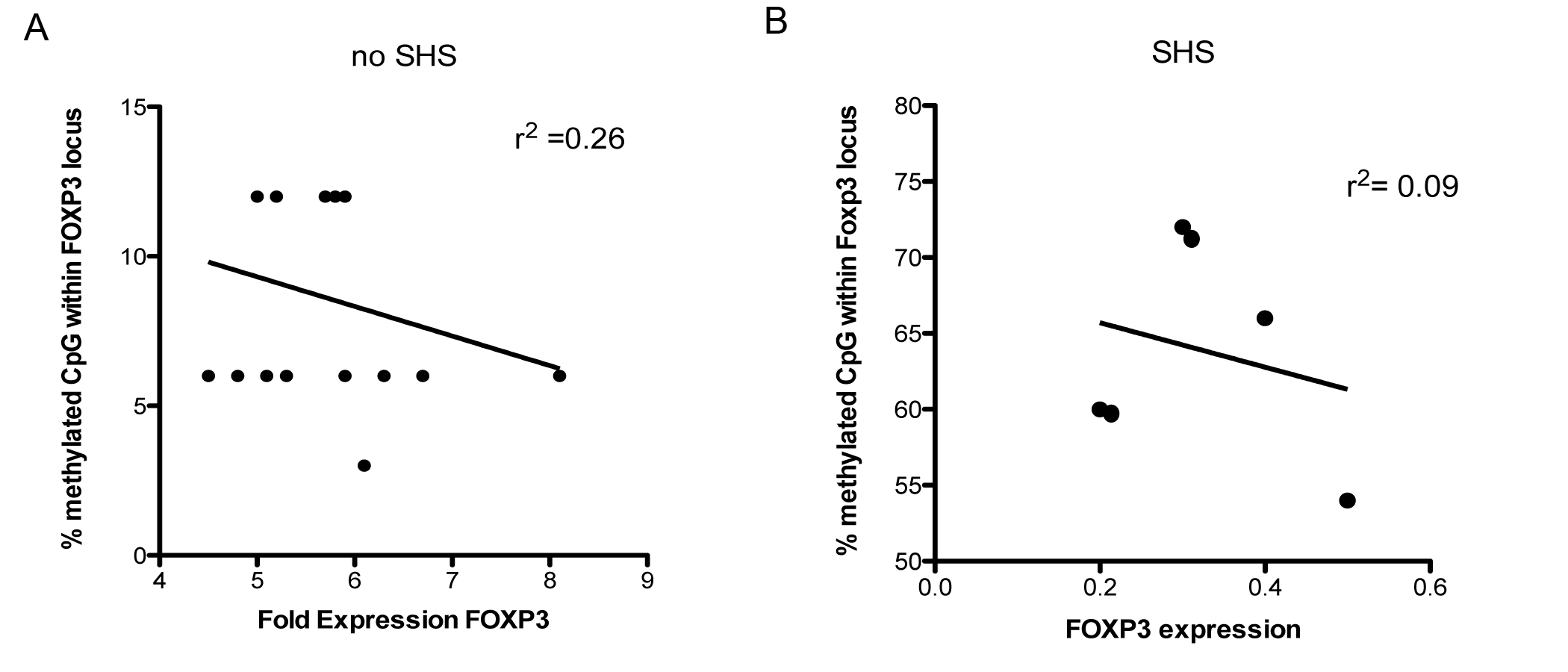

Supplement: Figure S2 — Linear regression analysis of FOXP3 expression and CpG methylation within the FOXP3 locus of asthmatics. A) Asthmatic MZT subjects with no SHS (n = 15) and B) All asthmatic MZT subjects with SHS exposure (n = 6). (TIF) [file pone.0048796.s002.tif]

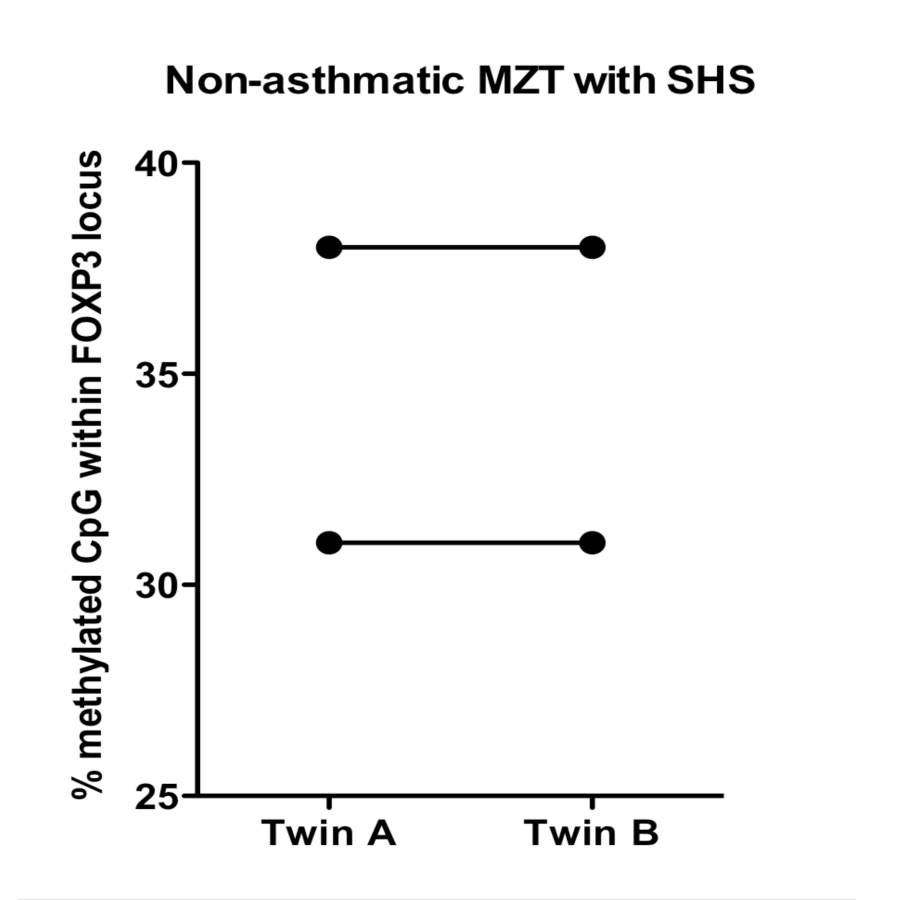

Supplement: Figure S3 — FOXP3 CpG methylation analysis in non-asthmatic MZT pairs with SHS (n = 2). (TIF) [file pone.0048796.s003.tif]
