# Supplementary material for: Asthma Discordance in Twins Is Linked to Epigenetic Modifications of T Cells
Source: PLoS One. 2012 Nov 30;7(11):e48796. doi: 10.1371/journal.pone.0048796 (PMC3511472; doi:10.1371/journal.pone.0048796)
Supplement: Data S1 — (DOC) [file pone.0048796.s004.doc]

**METHODS**

**Human subjects**

Criteria from the 2007 National Heart, Lung, and Blood Institute using clinical symptoms, signs, and forced expiratory volume in 1s (FEV_1_) was used to diagnose asthma. Patients with FEV_1_ <80% were classified as severe, those with FEV_1_ between 80% and 90% were classified as moderate, and those with FEV_1_ >90% and <100% were classified as mild. If only one twin used an inhaler and his or her FEV_1_ was lower than their healthy twin, this also was considered discordant. GIS (geographic information systems) was performed according to previously published methods[1]. The Principal Investigator of the Twin Research Registry at SRI International is Gary E. Swan, PhD, Director of the Center for Health Sciences. More information about the Registry can be obtained at http://www.sri.com/policy/healthsci/twin/.

**Functional Assays**

Assays were performed in round-bottom 96-well microtiter plates according to previously published methods (5) with fixed numbers of live cells per well for each cell type: CD4^+^ T cells, regulatory T cells, and antigen-presenting cells (APCs). In brief, autologous CD4^+^CD25^-^ conventional T cells, and autologous-irradiated CD3-depleted PBMCs (APCs) with or without autologous Treg cells, were added. In this way, determination of T eff proliferation and Treg suppression can occur. APCs were depleted of T cells by using StemSep human CD3^+^ T-cell depletion (StemCell Technologies), followed by irradiation with 40 Gy. All wells were supplemented with anti-CD3 mAb (clone HIT3a at 5.0 μg/mL). T cells are cultured for 7 days at 37°C in a 5% CO_2_-humidified atmosphere. Sixteen hours before the end of the incubation, 1.0 μCi ^3^H-thymidine was added to each well. Plates were harvested with a Tomtec cell harvester and ^3^H-thymidine incorporation determined by using a Perkin Elmer β-scintillation counter. Each sample was tested for 3 parameters: (1) Treg-cell anergy (Treg cell alone with APC); (2) Treg-cell suppression (CD4^+^ CD25^+^ Treg cell: CD4^+^T cell with APC (1:1)); and (3) T-cell proliferation (CD4^+^ T cell with APC). In some samples, tetanus toxoid (Sigma Aldrich) was added to the irradiated APC for 30 minutes at 37°C to assess Teff proliferative responses.

**CpG Methylation Site Analys**is

T cell subsets were purified and genomic DNA was isolated by using a DNA extraction kit (Qiagen). The DNA was denatured, modified with sodium metabisulfite, purified, and desulfonated by using a CpGenome Fast DNA modification kit (Chemicon International). DNA was then sequenced (SayoBiotech).

*Foxp3 Bisulphite-specific PCR and pyrosequencing.* Identification of *FOXP3* CpG loci of interest was based on our previous studies [11] of two regions where we found increased methylation of the 8 CpG islands in the promoter region and of the 13 CpG islands in the intronic regions. In this study, we sequenced a total of 13 CpG sites in the FOXP3 locus, 6 in the promoter region and 7 in the intronic region. Demethylation of these two regions was associated with stable *FOXP3* expression in memory Tregs. Partial methylation was associated with unstable *FOXP3* expression in Tregs. In contrast, the promoter and intronic regions of *FOXP3* in conventional CD4^+^ T cells are methylated. Detailed information about the primers used in the bisulphite-specific PCR are listed below.

CpG islands determined using NCBI gene searches, BLAST for sequence information; primers determined using PyroMark Assay Design Software (Qiagen).

**FOXP3 CpG sites:**

**FOXP3 primer design:**

|  | **promoter** | **intronic region** |
| --- | --- | --- |
| **Forward** | 5’-CAAATTCAGAGTATTAGTTCTTTTCCTCTTT-3’ | 5’-TTGGGTTAAGTTTGTTGTAGGAT-3’ |
| **Reverse** | 5’-[biotin] -GTGGCATGTCGCACCAAAAAGAAGAG -3’ | 5’-[biotin]-ACCCCCCACTTACCCAAATTTT-3’ |
| **Sequencing** | 5’-AAAGGCAAATTCA-3’ | 5’-GTAGGATAGGGTAGTTAG-3’ |
| **Description** | 6 CpG sites (-141, -95, -80, -66, -54, -27) | 7 CpG sites (+3951, +3956, +4105, +4224, +4228, +4236, +4245) |

*IFNγ Bisulphite specific PCR and pyrosequencing.* The detailed information of primers used in the bisulphite-specific PCR can be found listed below. For methylation analysis, five amplicons, amplified using a HotStar Taq kit (Qiagen), included a total of six CpG sites within the proximal promoter region of *IFNγ*. Direct quantification of methylated versus unmethylated cytosine nucleotides for each analyzed CpG site present in the amplicons was determined by pyrosequencing with the PSQ HS 96 Pyrosequencing System (Qiagen) and Pyro Gold CDT Reagents (Qiagen) as described previously. Positive methylation thresholds for each site were set at 70% of reactions or more. In each pyrosequencing assay, one amplicon was used for sequencing. Internal controls for bisulphite conversion efficiency were included in each pyrosequencing assay. A genomic sequence that is artificially methylated on all its CpG dinucleotides (Cat. # S7821, Millipore, Billerica, MA, USA) was also used in the bisulphite conversion, PCR, and pyrosequencing with the primers and sequencers mentioned above as a technical control.

**IFN-γ promoter region:**

-295bp -186 -54 “+1” 122,128 171

**IFNγ primer design:**

| **CPG Site*** | **Details** | **Sequences** |
| --- | --- | --- |
| –295 | Forward | 5′-[Biotin]TTTGTAAAGGTTTGAGAGGTTTTAGAAT-3′ |
|  | Reverse | 5′-CAAACCCATTATACCCACCTATACCA-3′ |
|  | Sequencer | 5′-TTTTATACCTCCCCACTT-3′ |
| –186 | Forward | 5′-TTAGAATGGTATAGGTGGGTATAATGG-3′ |
|  | Reverse | 5′-[Biotin] TATTATAATTAAAATTTCCTTTAAACTCCT-3′ |
|  | Sequencer | 5′-GGGTATAATGGGTTTGTT-3′ |
| –54 | Forward | 5′-GGGTTTGTTTTATAGTTAAAGGATTTAAGG-3′ |
|  | Reverse | 5′-[Biotin] AATCAAAACAATATACTACACCTCCTCTAA-3′ |
|  | Sequencer | 5′-TATTTTATTTTAAAAAATTTGTG-3′ |
| +122~ | Forward | 5′-[Biotin] TTTTGGATTTGATTAGTTTGATATAAGAA-3′ |
| +128 | Reverse | 5′-AAAACCCAAAACCATACAAAACTAAAA-3′ |
|  | Sequencer | 5′-CTAAAAAACCAAAATATAACTTAT-3′ |
| +171 | Forward | 5′-[Biotin] TTTTGGATTTGATTAGTTTGATATAAGAA-3′ |
|  | Reverse | 5′-CATTTTCAACCACAAACAAATACTATTAA-3′ |
|  | Sequencer | 5′-ACAACCAAAAAAACCC-3′ |

**QT-PCR**

RNA was isolated from purified T cell populations using RNeasy kits (Qiagen) according to the manufacturer's protocols. Similar amount of cells (200,000) were used for each subject. For cDNA synthesis, 500 ng of total RNA was transcribed with cDNA transcription reagents (Applied Biosystems) using random hexamers, according to the manufacturer's protocols. Gene expression of FOXP3 and IFNγ was measured in real time using primers and other reagents purchased from Applied Biosystems and SuperArray. All PCR assays were performed in triplicate. Data were presented as relative fold expression of the candidate gene to the expression of the housekeeping gene β glucuronidase.

References

1. Moore DK, Jerrett M, Mack WJ, Kunzli N (2007) A land use regression model for predicting ambient fine particulate matter across Los Angeles, CA. J Environ Monit 9: 246-252.

2. Nadeau K, McDonald-Hyman C, Pratt B, Noth B, Hammond K, Balmes J and Tager I (2010) Ambient air pollution impairs regulatory T-cell function in

asthma. J Allergy Clin Immun 126:845-852.
